# A Population Study of Relative Age Effects on National Tests in Reading Literacy

**DOI:** 10.3389/fpsyg.2019.01761

**Published:** 2019-08-07

**Authors:** Ole Petter Vestheim, Magne Husby, Tore Kristian Aune, Ottar Bjerkeset, Terje Dalen

**Affiliations:** ^1^Faculty of Education and Arts, Nord University, Bodø, Norway; ^2^Faculty of Nursing and Health Sciences, Nord University, Bodø, Norway

**Keywords:** month of birth, maturation, gender differences, assessment, school attainment

## Abstract

We examined relative age effects (RAE) in national test results in reading literacy in Norway in 2013, in Grades 5, 8, and 9 students (*n* = 173,421) to estimate how month of birth is associated with mean scores and different achievement levels. The results confirm that there is an approximately linear decrease in test scores across months of birth for all grades and both genders. Consequently, students born early in the year are more likely to end up at higher achievement levels than students born later in the year. Possible explanations for this phenomenon are the greater maturity of older children and that they might be considered more gifted when compared with their younger peers. Further, we found increasing gap in test scores between girls and boys from grade 5 to 8 and 9. We suggest both maturity and motivational reasons for these differences.

## Introduction

During the last few decades, there has been increased focus on testing students’ school achievement in several subjects and basic skills. Transnational tests like PISA, PIRLS, and TIMMS have been developed to measure learning outcomes (Hopfenbeck, 2014). In 2004, national tests were introduced in Norway as part of the national quality assessment system. National tests in Norway are held in fifth, eighth, and ninth grades, and test the basic skills of reading literacy, numeracy, and the English language. Results from these tests give an opportunity to explore students’ achievement and how they might be affected by month of birth. All students, except in some special cases, are supposed to participate in the national tests. The purposes of introducing national tests is to assess if schools manage to develop and increase students’ basic skills (Blömeke and Olsen, 2018), and contribute to information about student learning so that people engaged in practice can support their students’ progress (Vestheim and Lyngsnes, 2016). The Ministry of Education and Research is ambiguous about what information the test results provide. On one hand, it is claimed that the results give valuable information about groups of students (school and municipality levels), but not detailed enough information about individual students. One the other hand, national tests are supposed to be used to inform both students and their parents about the students’ achievement to be able to follow up their study progression (Norwegian Directorate for Education and Training, 2011, p. 70–73). Hence, it is important for policymakers, school leaders, and teachers to understand what these test scores mean and how different variables impact the test results.

Student admission into the primary school system is often determined solely by using birth date as a cut-off, and in Norway this is January 1st. Consequently, all children born in the same calendar year start at the same time, yet the school year starts in August and ends in June. This means that the age difference in one single class or cohort can vary by up to a year (Aune et al., 2018). Studies have repeatedly found that differences in month of birth have a significant influence on academic achievement. Students who are younger tend, consistently throughout compulsory education, to score lower on tests of academic ability than their older peers (Daniels et al., 2000; Crawford et al., 2007, 2011; McEwan and Shapiro, 2008). These differences are often referred to as the “birthday effect,” “month of birth effect,” “age at school entry effect,” “season of birth effect,” and/or “relative age effect” (Angrist and Keueger, 1991; Musch and Grondin, 2001; Robertson, 2011; Ponzo and Scoppa, 2014). Studies have found that relative age effects (RAEs) are both systematic and persistent in the schooling system (Jinks, 1961; Cobley et al., 2009; Aune et al., 2015, 2018; Dalen et al., 2017). More specifically, relatively younger children achieve lower scores and more often have special needs, including special education (Wallingford and Prout, 2000; Cobley et al., 2009). Further, the RAE in test achievement seems to be consistent across school subjects and RAEs seem to persist throughout the education process (Sharp et al., 1994; Massey et al., 1996). Nam (2014) found that monthly differences in age had a significant effect on academic achievement until middle school among Korean students. Sprietsma (2010) evaluated the long-term causal effect of relative age in the first grades of primary school on pupil tests, using PISA 2003 scores as data, and found that an older age than one’s peers was linked to better results in both reading and mathematics. In keeping with this (Robertson, 2011, p. 305), reported that pupils born at the end of the year achieved lower scores on both math and reading exams. In their study, third graders born in the 9 last months of the year scored about 20% of a standard deviation lower than those born in the first 3 months (Robertson, 2011). The study also concludes that these within cohort effects are similar to or greater than the effects of race, gender, and parental income on students’ testing achievements. Results from three different data sets for 9-, 13-, and 15-year-old students (PIRLS, 2006, TIMMS, 2007; PISA, 2009) and the work of Ponzo and Scoppa (2014) all confirm that school entry age has an effect on the test scores in these tests. Younger children score substantially lower than older peers at the fourth, eighth, and tenth grade.

Relative age effects is at its maximum when a child born December 31st is compared with another child born January 1st the same year. Although RAE seem to operate into adulthood in terms of higher test scores and level of education (Kawaguchi, 2011). It is reasonable to expect that this effect will start decreasing already in childhood and the adolescent years, as students all reach maturity and the relative age difference gradually diminishes (Pehkonen et al., 2015). Generally, adults often have higher expectations of children born early in the year and they are therefore more often defined as talented or gifted (Musch and Grondin, 2001). RAE in school tests might therefore be further enhanced by the fact that student performance will increase when the expectations are higher (see Rosenthal, 1987). This is in line with Harter’s (1978) competence motivation theory suggesting that a person’s motivation increases when the person successfully mastering a task. Further, Wigfield (1997) further suggests that children’s reading motivation relates to both reading performance and frequency.

In a review of 118 studies, Court (1983) concluded that performance in general intelligence test did not differ between the genders. Another meta-analysis support this finding among children aged 6–14 years, yet reported that males obtain higher mean scores from the age of 15 through to old age (Lynn and Irwing, 2004). This view was challenged by the cognitive maturity hypothesis, which assumes that gender differences in physical maturity at the end of primary school are linked to gender differences in cognitive maturation and, thereby, differences in achievement in subjects at school (Colom and Lynn, 2004). This hypothesis suggests that girls have the fastest increase in IQ points during their early teens and boys have the fastest increase later in their teens. Gender differences in IQ are perceived as an indicator of a comprehensive social maturation process that gives girls a greater assessment ability than boys and, thereby, makes it easier for girls to adapt to school requirements such as work habits and independent work (Colom and Lynn, 2004; Reynolds et al., 2008). With increasing cognitive skills, students will also increasingly endure school evaluation pressure and become less reliant on a stimulating learning environment to perform (Reynolds et al., 2008; Lindberg et al., 2010). Brizendine (2006) reported inequalities in girls’ and boys’ brains that can be linked to language development and ability to perceive and use language. Others report that girls’ brains develop on average faster than boys, making them susceptible to complex information earlier and generally makes it easier for girls to concentrate (Gurian, 2010).

Even though the maturity hypothesis points to gender differences in achievement, it is reasonable that the maturity hypothesis can also explain why students born earlier achieve higher results than students born later in the same year.

Data from national tests in Norway include all students in Grades 5, 8, and 9, and therefore offer a good basis to study the RAE. Available data for three grades also provides an opportunity to examine if or to what extent the RAE might operate from childhood to the adolescent years. The basic skills of reading literacy are given a high priority in the Norwegian educational system and the aim of the present study is to explore potential RAEs in the national tests in reading literacy. We formulated the following specific objectives: (1) *Is there a RAE in national tests in reading literacy in Norway, and if so*, (2) *how does the RAE change from Grade 5 to Grade 8 and 9*, and (3) *are there any gender differences in RAE?* It is hypothesized that (1) a RAE exists and (2) declines with age. It was also expected to (3) find gender differences. In addition, the study intended to explore how potential gender differences might change from Grade 5 to 9.

## Materials and Methods

### Participants

The Norwegian Directorate of Education and Training was responsible for data collection in this study. The data represent a complete set of scores from all students who participated in national tests held in the basic skills of reading literacy in 2013 (*N* = 173,421). For Grade 5, we had data on 55,464 students (27,807 girls and 27,657 boys, monthly birth rates ranging from 4,096 to 5,038) For Grade 8, the dataset included 59,105 students (29,117 girls and 29,988 boys, monthly birth rates from 4,302 to 5,393), and for Grade 9, 58,852 students (29,825 girls and 29,027 boys, monthly birth rates from 4,285 to 5,377). About three percent of the students were exempted from the test because of special needs and 0.4% did not participate for other reasons. The data provided by The National Directorate of Education and Training included information on the month of birth, grade, gender, and final score in the national test on reading literacy.

The study was conducted according to the Helsinki Declaration and has been approved by the Norwegian Social Science Data Services (NSD). The data was collected and delivered to us by The Norwegian Directorate of Education and approved according to national regulations. The study is based on a secondary analysis of data and therefore further parental consent was not required.

### Instruments

Students were tested in Grades 5, 8, and 9. The maximum test score was 33 points in fifth grade, and 48 points for tests held in the eighth and ninth grades (same tests were used in Grades 8 and 9). Students are placed in achievement levels from 1 to 3 in the fifth grade, and 1 to 5 in the eighth and ninth grades (Table 1).

**TABLE 1:** Overview of test scores and their respective achievement levels (1–5).

	**Level 1**	**Level 2**	**Level 3**	**Level 4**	**Level 5**
Grade 5	0–16	17–27	28–33		
Grades 8 and 9	0–14	15–23	24–34	35–40	41–48

### Procedure and Analyses

National tests in reading literacy are carried out every year in Norway. The test is given in the first semester in Grades 5, 8, and 9 on the same date, set by The National Directorate of Education and Training. We carried out analyses to explore how the relative age effect operates and develops from fifth to eighth and ninth grades. Secondly, we examined how students’ birth months are reflected in achievement levels.

### Statistics

Kolmogorov–Smirnov tests, histograms, and Q–Q plots were applied to confirm normality assumptions of the variables. The effect of birth month and gender on standardized test scores were examined with GLM univariate ANOVAs separately for Grade 5, 8, and 9. In the GLM univariate ANOVAs pairwise comparisons, the alpha was Bonferroni corrected and the partial eta squared (η^2^p) was applied as a measure of effect size, where 0.01 < η^2^p < 0.06 constitutes a small effect, 0.06 < η^2^p < 0.14 constitutes a medium effect and η^2^p < 0.14 constitutes a large effect (Cohen, 1988). A Generalized Linear Model (GLZ) in SPSS (IBM SPSS Statistics 24) was used, with test score as a dependent (response) variable, and month of birth (1–12) and gender (1–2) as independent variables (predictors). The independent variables were compared with intercept. GLM is a flexible generalization of ordinary linear regression that allows for response variables that have error distribution models other than a normal distribution. We assumed a Poisson log linear distribution of the dependent variable in the model and used the SPSS default settings in the analyses, except for the estimated model means for which contrast difference were selected. We analyzed students’ results in Grades 5, 8, and 9 separately using a logistic regression analysis. The test of model effect confirmed that the logistic regression model had good fit. In terms of descriptive statistics, unadjusted mean test scores and prevalence estimates of students on each achievement level was used to communicate the observed trends.

## Results

**FIGURE 1:**
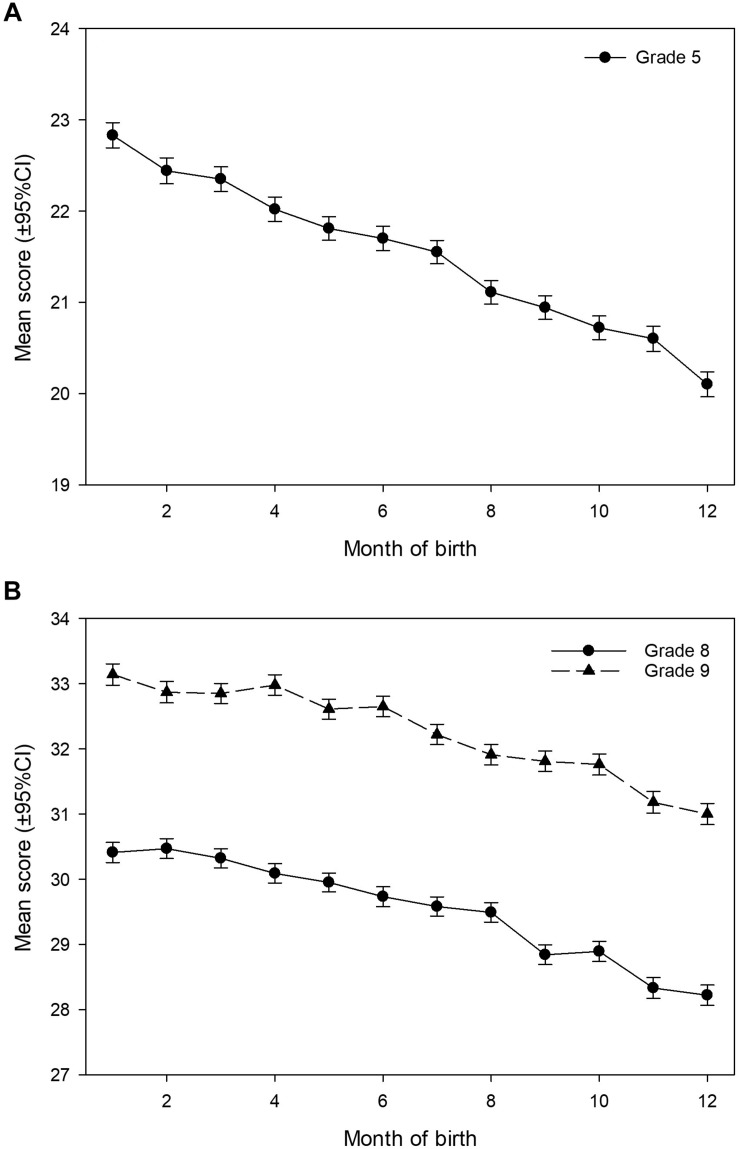
Mean scores in the national reading test for Grades 5 **(A)** and 8 and 9 **(B)** relative to month of birth, with 95% Wald confidence intervals.

The results showed that both month of birth and gender significantly influenced the score in reading skills. As could be expected the difference in test scores between birth months were highest in Grade 5 [*F*(1,11) ≥ 69.6, *p* < 0.001, η^2^p ≥ 0.014] and lower in Grade 8 [*F*(1,11) ≥ 35.9, *p* < 0.001, η ≥ 0.007] and Grade 9 [*F*(1,11) ≥ 30.0, *p* < 0.001, η ≥ 0.006]. Contrary to this, the difference between gender where lower in Grade 5 [*F*(1) ≥ 93.1, *p* < 0.001, η^2^p ≥ 0.002] and highest in Grade 8 [*F*(1) ≥ 1054.0, *p* < 0.001, η ≥ 0.018] and Grade 9 [*F*(1) ≥ 1395.7, *p* < 0.001, η ≥ 0.023]. Students born in December had the lowest scores when corrected for gender, and lower scores than all other months for Grade 5 and all other months except November for Grades 8 and 9 (Table 2). The model calculated sound statistical evidence for a difference in score from each month to the previous month in all of the eleven pairs of tests between two consecutive months for Grade 5, and in ten of the eleven tests in Grade 8 and 9. The gradual decline in score for students born later in the year is shown in Figure 1.

**TABLE 2:** Model parameter estimates (logit scale) from the logistic regression analysis with test scores for Grades 5, 8, and 9 as dependent variables, respectively, and month of birth and gender as independent variables^1^.

	**Intercept**	**January**	**February**	**March**	**April**	**May**	**June**	**July**	**August**	**September**	**October**	**November**	**Gender (boy)**
Grade 5 (B±SE)	3.013 ± 0.004	0.128 ± 0.005	0.110 ± 0.005	0.106 ± 0.005	0.091 ± 0.005	0.082 ± 0.005	0.077 ± 0.005	0.070 ± 0.005	0.049 ± 0.005	0.041 ± 0.005	0.031 ± 0.005	0.025 ± 0.005	−0.025 ± 0.002
Grade 8 (B ± SE)	3.381 ± 0.003	0.075 ± 0.004	0.077 ± 0.004	0.072 ± 0.004	0.064 ± 0.004	0.060 ± 0.004	0.052 ± 0.004	0.047 ± 0.004	0.044 ± 0.004	0.022 ± 0.004	0.024 ± 0.004	0.004 ± 0.004	−0.082 ± 0.002
Grade 9 (B ± SE)	3.476 ± 0.003	0.067 ± 0.004	0.058 ± 0.004	0.058 ± 0.004	0.062 ± 0.004	0.051 ± 0.004	0.052 ± 0.004	0.039 ± 0.004	0.029 ± 0.004	0.026 ± 0.004	0.024 ± 0.004	0.006 ± 0.004	−0.085 ± 0.002

*^1^The independent variables are compared with intercept, which represents month 12 of birth (December) and gender 2 (girl). Df=1 in all tests. In all tests, *p* < 0.001, except for November in Grades 8 and 9, which were non-significantly different from December. SE, Standard error.*

There was a tendency for the influence of month of birth to decline, while the influence of gender increases, by increasing student age (Table 2). The difference in score between the genders was small for fifth grade. The estimated score was 21.77 ( ± 0.028 SE) points for girls and 21.33 ( ± 0.028) points for boys corrected for month of birth. Overall, girls obtained a 2% higher score than boys (Table 2). For eighth grade, girls obtained at average 30.75 ( ± 0.031) points and boys 28.34 ( ± 0.033) points when corrected for month of birth – an 8.5% difference (Table 2). For ninth grade, girls obtained at average 33.64 ( ± 0.034) points and boys 30.90 ( ± 0.032) points when corrected for month of birth – an 8.9% difference (Table 2).

**FIGURE 2:**
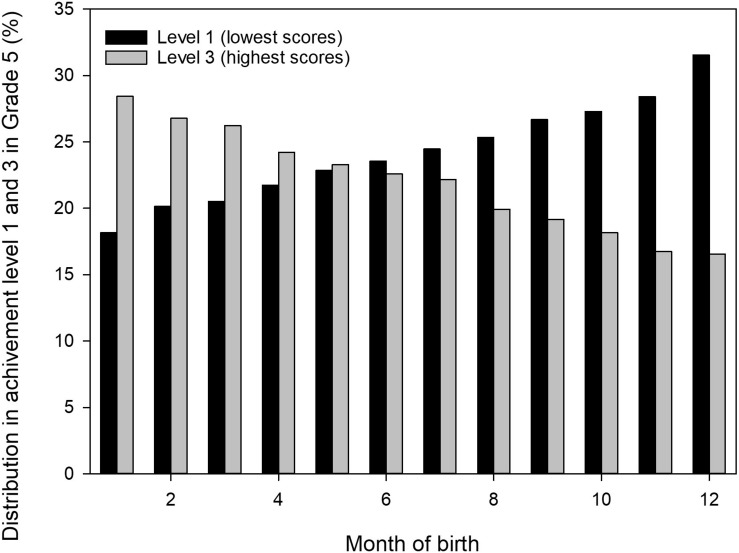
Percentage distribution of Grade 5 pupils in achievement level 1 (lowest scores) and achievement level 2 (highest scores) for each month of birth.

There was a 13% difference in mean test score results between students born in January and December in the total sample. Consequently, the proportion of students ending up in Achievement Level 1 (low achievement) is higher for students born late in the year than their older peers; (high achievement; see Figure 2).

## Discussion

This national study of nearly 175,000 Norwegian students confirms a consistent RAE in reading literacy testing through Grades 5, 8, and 9. In keeping with the literature, reading literacy is highest among the oldest pupils in each grade and RAE decreased from Grade 5 to Grade 9. Consequently, birth month was also closely linked to the estimated individual achievement level. Further, we found an increasing gap in test scores between girl and boys from Grade 5 to 8 and 9.

### Mechanisms Explaining Relative Age Effects in Education

Students maturity generally reflects advantages in readiness for intellectual development (Reynolds et al., 2008; Lindberg et al., 2010). The psychological consequences of this advantage in the earlier born may also contribute to higher confidence and self-esteem compared to younger and cognitively less mature students in the same grade (Cobley et al., 2008). Conversely, younger students might internalize unfair comparisons and believe themselves to be less capable. Harter’s (1978) competence motivation theory suggests that students who consider themselves as gifted are more likely to continue the process of developing their abilities and put more time and effort in the subject they master. Since students born early in the year often perceive themselves as more competent than peers born later in the year As a result, one could expect that Grade 8 students who were born early would achieve higher test scores than those Grade 9 students who were born late. However, this was not found in our study. This indicates that students’ test score results can mainly be explained by age at test, rather than other explanations.

Other mechanisms causing the relatively higher test scores differences for students born earlier than those born later in the year cannot be directly tested in this dataset. From sports literature, physical differences among children at different ages are often cited as being a significant contributor to higher rates of success of relatively older players (Musch and Grondin, 2001). This might be a less reliable explanation for test scores in school, where physical prowess is not as directly necessary for achievement in an academic skill such as reading literacy. However, another explanation proposed in this study for RAE might be that the oldest students have greater – up to 1 year – reading experience before school enrollment. Research suggests that the quantity and quality of practice are basic mechanisms which, at least partly, explain skill and achievement level (Baker and Horton, 2004).

### Gender Differences in Test Scores

Our results indicate increasing gender differences in achievement between boys and girls from Grade 5 to 9. This development might be explained by an earlier onset for girls in developing cognitive skills and higher IQ, as suggested in the cognitive maturity hypothesis (Colom and Lynn, 2004; Reynolds et al., 2008). Another explanation can be that girls generally are more devoted to school and thereby put more effort into these tests. There is also a consistent finding in the literature that girls have a more positive attitude to recreational reading than boys (see Logan and Johnston, 2009), which might become apparent in the national tests. Regardless of the mechanisms explaining this phenomenon, we recommend that future studies examine possible gender-specific trajectories of RAE further into adulthood.

### Use and Interpretation of National Tests in School

The intention of the national tests in Norway was never to label the students with grades; rather, these tests are meant to help the teacher to adapt their teaching so that every student can reach their maximum potential in reading. On an individual level, however, national test scores will probably influence the teacher – student relation, as the test outcome and the gained achievement level can lead to lower or higher expectations from the teachers. Alternatively, low scorers might get even more help in improving their ability in reading.

On a societal level, Norway is considered as a low-stake country when it comes to consequences of testing and test results. This means that high or low scores on, in this case National tests in reading, does not have any direct influence for either the students, teachers, schools or the municipality as school owners. In some cases, schools can be exposed for naming, shaming, and blaming, or opposite, in media because of high- or low-test scores. In high stake testing regimes results are followed by incentives and can have consequences for students, teachers, school leaders, and schools. The national tests in Norway are primarily meant to be a formative assessment tool for policymakers, school owners and teachers to both control and adjust teaching practices with the aim of improving all student’s achievement in reading as a basic skill. Given the clear evidence of RAE in school testing, teachers who choose to use these test results for individual student and parent feedback should always interpret the scores with the students age and birth month in mind.

## Conclusion

The present study confirmed a RAE in achievement in national tests in reading literacy for Grades 5, 8, and 9 (hypothesis 1) Mean reading scores decreased systematically with birth month in each grade (hypothesis 2), and this applies to both genders. The test scores continue to decrease if scores from Grades 8 and 9 are combined over 2 years. This indicates that students’ test score is mainly related to age at test, rather than other explanations. In addition, an increase in gender differences from Grade 5 to 8 was observed. If national tests are used to label students at different achievement levels, it is important that people engaged in the educational system are aware of the relative age effect. Otherwise, there is an undesirable risk that students born late could be labeled as less gifted than their older peers, solely based on their younger age and maturity level.

## Data Availability

The datasets for this manuscript are not publicly available because the data is owned by the Norwegian Educational Government. Requests to access the datasets should be directed to ole.p.vestheim@nord.no.

## Ethics Statement

The data for this study was collected by, and received from The Norwegian Directorate of Education in a de-identified format and approved according to the national regulations. The study reported in this manuscript is a secondary analysis of this data and has been approved by the Norwegian Social Science Data Services (NSD). The data used did not contain any identifiers and a minimum of variables (birth month, gender, test score, and grade). Further approval by an Ethics Committee and/or consent was not required as per the NSD and applicable national regulations.

## Author Contributions

All authors listed have made a substantial, direct and intellectual contribution to the work, and approved it for publication.

## Conflict of Interest Statement

The authors declare that the research was conducted in the absence of any commercial or financial relationships that could be construed as a potential conflict of interest.
